# Synthesis of Super-Long Carbon Nanotubes from Cellulosic Biomass under Microwave Radiation

**DOI:** 10.3390/nano12050737

**Published:** 2022-02-22

**Authors:** Joy Esohe Omoriyekomwan, Arash Tahmasebi, Jian Zhang, Jianglong Yu

**Affiliations:** 1 Key Laboratory of Advanced Coal and Coking Technology of Liaoning Province, School of Chemical Engineering, University of Science and Technology Liaoning, Anshan 114051, China; joyomoriyekomwan@yahoo.com (J.E.O.); zhangjian7216599@163.com (J.Z.); 2 Department of Chemical Engineering, University of Newcastle, Callaghan, NSW 2308, Australia; arash.tahmasebi@newcastle.edu.au; 3 Monash Research Institute of Science and Technology (Suzhou Industrial Park), Southeast University—Monash University Joint Graduate School, Suzhou 215000, China

**Keywords:** super-long carbon nanotubes, microwave radiation, carbon materials

## Abstract

This study reports a novel method for synthesizing super-long carbon nanotubes (SL-CNTs) from cellulose via a microwave treatment process without an external catalyst. CNTs with a length of 0.7–2 mm were obtained via microwave treatment of cellulose biochar temperatures of 1200–1400 °C. Scanning electron microscope (SEM), together with high-resolution transmission electron microscope (HRTEM) results, were used to investigate the changes in the length and morphology of CNTs with respect to treatment temperature. The morphology of CNTs changed from twisted, curved, and threadlike to straight structures. The average length of CNTs after microwave pyrolysis at 600 °C was approximately 600–1800 nm, which after microwave treatment at 1300 °C and 1400 °C increased to about 1–2 mm. X-ray diffractometer (XRD) results confirmed the crystalline structure of CNTs with two prominent peaks at 2θ = 26.3° and 2θ = 43.2° correlating with the graphite (002) and (100) reflections. The I_D_/I_G_ ratio obtained from Raman spectra of the CNTs decreased to the lowest value of 0.84 after microwave treatment at 1400 °C, implying a high degree of carbon order. The presence of Fe and trace amounts of other elements were confirmed by the energy-dispersive X-ray spectrometer (EDS) and were postulated to have catalyzed the growth of CNTs. The mechanism of the SL-CNTs growth under microwave treatment was proposed and discussed.

## 1. Introduction

After their discovery in 1991 [1], carbon nanotubes (CNTs), one of the many allotropic forms of carbon, have been extensively studied. They are known as unique materials due to their exceptional properties, such as mechanical strength, durability greater than steel, electrical conductivity better than copper, and low density, about four times lower than these materials [2]. The use of CNTs in various electrical, lightweight, and high-strength applications is advantageous due to their unique properties, such as high tensile strength and high modulus, as high as 150 GPa and a 1 TPa, respectively [3,4,5]. Despite these unique properties, carbon nanotubes still have significant drawbacks, such as small dimensions and high cost, and non-sustainable synthesis methods that hinder their full potential [2].

Carbon nanotubes have been successfully synthesized using various methods, such as laser ablation [6], chemical vapor deposition (CVD) [2,7,8,9], arc-discharge [10], catalytic chemical vapor deposition (CCVD), and plasma-enhanced chemical vapor deposition (PECVD) [3,11,12]. These methods usually necessitate using metallic catalysts (e.g., Ni, Mo, Fe, Co, etc.) [13,14,15], substrate [16], and hydrocarbons as carbon sources (e.g., ethane, methane, acetylene, xylene, or a mixture of these) [17]. Generally, catalyst deactivation during the synthesis of CNTs limits their growth and subsequently hinders their practical application [2]. This can be overcome by developing a novel method for synthesizing long CNTs to improve their unique properties to realize their full potential for industrial applications. In some applications, the electrical and mechanical properties of CNTs can only be harnessed when they are long; such structures can be used as electrochemical microactuators or stable, highly conducting micro-cables [18].

Super-long carbon nanotubes (SL-CNTs) have gained increasing attention for numerous plausible applications, including sensors [19,20], nanoelectronics [21,22] and multifunctional composites [23,24]. The more extended structures improve the thermal and physical properties [25,26]. The length of CNTs usually varies in orders of a few tens of nanometers to millimeters, depending on the synthesis conditions. The length of CNTs is an essential parameter in various applications. For instance, the performance of electronic devices and the generation of conductive networks are determined by nanotube length. In applying nanotubes in composite materials, the length of the nanotubes also determines the load transfer efficiency.

The synthesis of SL-CNTs remains a challenge. There have been some attempts by other researchers to produce SL-CNTs. Chakrabati et al. [27,28] synthesized 5-mm-long aligned CNTs. The super-long CNTs were synthesized through water-assisted CVD using Si/SiO_2_ wafer, Fe catalyst, at a pressure of 1 atm, with helium/H_2_ (2.5:1 *v*/*v*) as the carrier gas. The carbon source was high-purity (99.99%) ethylene. SL-CNTs were synthesized at 700 °C for about 10 h. The CNTs were generated due to the super-saturation of a carbon-metal solution, which induced carbon segregation leading to CNT formation from the catalyst surface. Loiseau and Pascard [29] used the arc-discharge method to synthesize long CNTs. They studied fourteen elements (3d and 4f elements), and it was discovered that long nanotubes were produced when Se, S, Sb, and Ge were used. They related the mechanism to the oxidation and electronic state of the elements. Cho et al. [30] used the CVD process to grow long vertically aligned CNTs using an Al_2_O_3_ catalyst. They proposed the root growth mechanism based on the carbon source and real-time photography analysis and observed that the length of CNTs was time and temperature dependent. Ghemes et al. [2] synthesized long, multiwalled carbon nanotubes via the CVD method using iron carbide. They reported that CNTs formed during iron carbide nucleation into the catalyst nanoparticles, while the length of CNTs depended on catalyst nanoparticles’ temperature and stability.

Despite the recent achievements in the synthesis of SL-CNTs, the cost remains high due to the complexity of the synthesis methods. Moreover, methods of synthesis of SL-CNTs are not sustainable due to the use of carbon source gases derived from fossil fuels. Further, there is no report on the production of super-long carbon nanotubes (SL-CNTs) from biomass. Biomass is a natural carbon energy source and has gained increasing attention as a precursor [31] to synthesize carbon materials [32,33], such as carbon nanotubes [34]. This study reports a novel and sustainable method of synthesizing super-long carbon nanotubes (SL-CNTs) from biomass under microwave treatment. Microwave-assisted synthesis has been reported to provide efficient and selective heating arising from electromagnetic energy conversion into thermal energy on a molecular scale. This novel method does not require any external catalysts or carbon sources. Various standard techniques were used to investigate the composition and structure of the super-long nanotubes and to propose their mechanisms of formation and growth.

## 2. Materials and Methods

### 2.1. Materials

Cellulose derived from palm kernel shell (PKS) biomass was used as the feedstock in this study. PKS, purchased from Malaysia, was ground into a size fraction of 150–280 µm and subjected to the cellulose isolation method. The palm kernel shell sample was composed of 33.04 wt.% cellulose, 45.59 wt.% lignin, 23.82 wt.% hemicellulose, and extractives 9.89 wt.% [35]. The chemical used and the step-by-step isolation method of PKS components have been reported in our previous study [35]. After isolation, ASTM standards for α-cellulose (ASTM D 1103-60) were used to verify the cellulose composition. The cellulose bio-component was oven-dried for 12 h at 80 °C to eliminate moisture. Activated carbon (AC) was used as the microwave receptor since PKS biomass is a poor microwave absorber. The particle size of the AC used was in the range of 300–600 µm for easy separation by sieving from cellulose after microwave treatment.

### 2.2. Microwave-Induced Synthesis of SL-CNTs

In the first step, CNTs were synthesized from the cellulose sample under fast microwave pyrolysis. Cellulose pyrolysis was conducted in a temperature-programmed microwave oven (Tangshan Microwave Thermal Instrument CO. Ltd., Tangshan, China), operating at a frequency of 2.45 GHz and maximum power output of 2000 W. The cellulose sample was mixed with a microwave receptor (activated carbon) at the ratio of 10:2 to reach the desired pyrolysis temperatures of 600 °C. The mixture of biomass and AC was loaded into a quartz reactor and placed in the microwave oven. A thermocouple was placed inside the reactor to monitor the sample temperature at 2 s intervals. High purity nitrogen gas (99.999%) at 400 mL/min flow rate was purged through the system for at least 30 min to eliminate air before experiments in a N_2_ atmosphere. Microwave pyrolysis runs were carried out for 30 min. After the pyrolysis, the cellulose char, which contained CNTs, was collected after separation from the AC by sieving.

In the second synthesis step, high-temperature microwave treatment was used to obtain SL-CNTs. About 5 g of cellulose char was loaded in a ceramic crucible and placed into a larger custom-made quartz reactor (high-temperature resistant). A high-temperature thermocouple was placed inside the reactor to monitor the sample temperature. At 400 mL/min, nitrogen gas (99.999%) was purged through the system for at least 20 min to eliminate air before experiments in the N_2_ atmosphere. Microwave treatment runs were conducted for 30 min at the desired temperatures of 1200 °C, 1300 °C, and 1400 °C.

### 2.3. Characterization of Carbon Nanotubes

The surface morphology of CNTs was studied using scanning electron microscopy (SEM, ZEISS Sigma HD, Oberkochen, Germany). The image measurement was obtained using an in-lens detector at an energy of 2 kV to achieve a clear image (clear visualization). The CNTs were also analyzed with a high-resolution transmission electron microscope (HRTEM) (Fei Tecnai F20) equipped with energy-dispersive X-ray spectroscopy (EDS). The system was set at an accelerating voltage of 120 kV, and images were acquired digitally by a high-resolution camera. The obtained images were processed with digital micrograph [36,37]. The length of the CNTs was measured with ImageJ software using direct imaging from the SEM. Image-processing software such as ImageJ has been used to estimate particle length. This software provides accurate length and diameter measurements [38]. The CNT size and length range of the CNTs were obtained from different particles. The diameter of the CNTs was also measured. The composition and carbon microstructure of the CNTs were also analyzed using a high-resolution transmission electron microscope (HRTEM) (Fei Tecnai F20) equipped with energy-dispersive X-ray spectroscopy (EDS). Rietveld refinement quantitative analysis was used to analyze the crystallinity of biochar. The Rietveld method entails quantification analysis established by an internal crystalline standard. The X-ray powder diffraction (in this case, silica powder) is used to estimate amorphous phase content. The peak shape formations, microstructural parameters, peak shape formations, crystal structure information, and background contribution were used to determine the diffraction profile. The crystalline phase of the weight fraction was calculated using Equation (1):

(1) $$w_{{\dot{i}}_{,}c}=w_{i}\frac{w_{s,w}}{w_{s}}\left(\frac{1}{1-w_{s}}\right)$$

where *w_i_* and *w_s_* represent refined weight portions of phase *I* and the internal standard, respectively, *w_i,c_* symbolize the original weight portions of phase *i*, and *w_s,w_* depicts the initial weight of the internal standard. The composition of the inorganic matter in cellulose was analyzed using X-ray fluorescence (XRF) (Thermo Fisher Scientific, Zurich Switzerland), and the results are shown in Table 1. The PKS cellulose contained high amounts of Ca, Fe, and Si and trace amounts of other elements. The crystalline structure of CNTs was investigated with a Rigaku Ultima IV X-ray diffractometer (XRD, Japan). Raman spectroscopy fitted with a laser diode of 532 nm (Horiba Jobin Yvon Xplora plus, Horiba Scientific, Shanghai, China) was used to study the changes in carbon ordering of CNTs with respect to temperature. The Raman spectra of samples were recorded in a wavenumber range of 500–3500 cm^−1^ with an acquisition time of 3 min.

## 3. Results and Discussion

### 3.1. Morphology and Microstructure of Carbon Nanotubes

**Figure 1:**
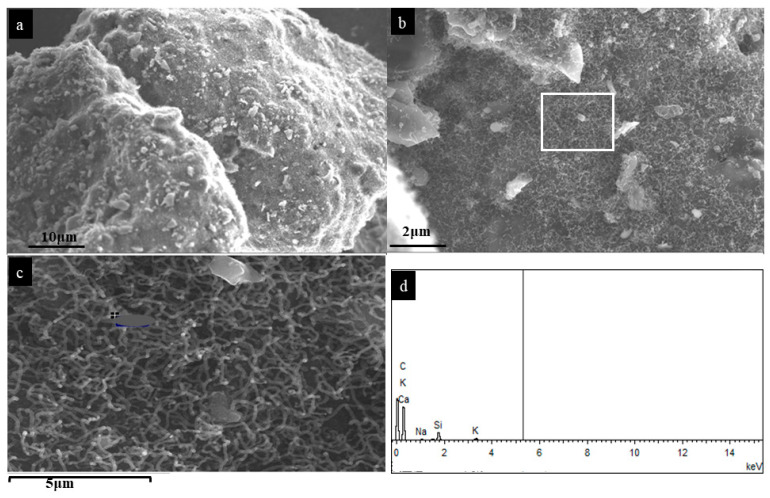
SEM of (**a**) grown CNTs at 600 °C, (**b**) magnified image, (**c**) magnified image of marked section in (**b**), (**d**) EDS spectra from (**c**) image.

Figure 1 shows the SEM images of the CNTs formed at 600 °C. The CNTs exhibited twisted, curved, and threadlike morphology on the cellulose char particles. The length and diameter of CNTs were in the range of 600–1800 nm and 50–100 nm, respectively. The EDS results displayed in Figure 1d revealed high carbon content in the CNTs, and trace amounts of Si, Ca, K, and Na. The inorganic matter was believed to have originated from cellulose since no external material was used in the process.

**Figure 2:**
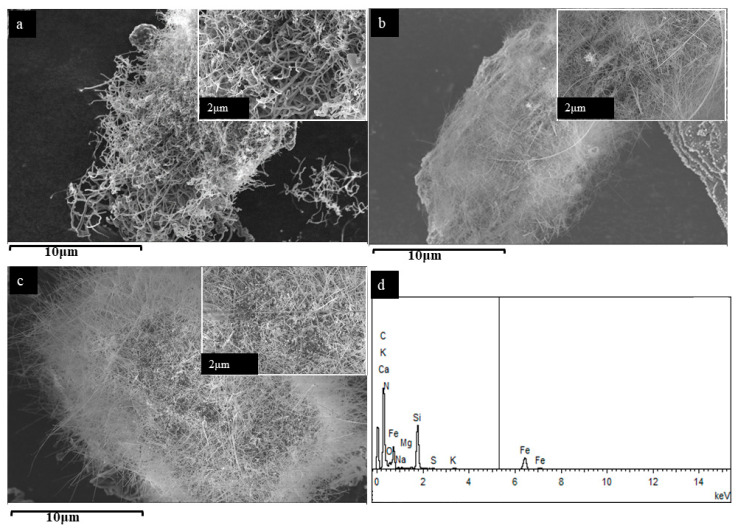
SEM images of SL-CNTs after microwave treatment: (**a**) 1200 °C, (**b**) 1300 °C, (**c**) 1400 °C, (**d**) EDS spectra of SL-CNTs prepared at 1300 °C. Magnified images (insets; top right).

**Figure 3:**
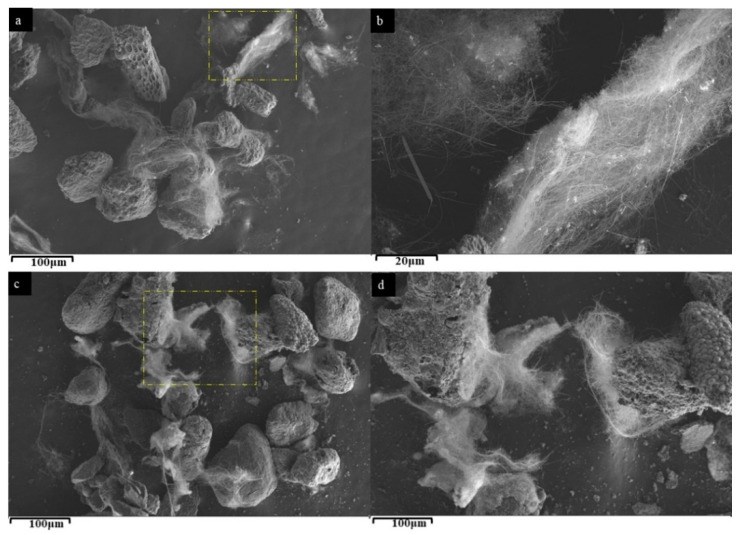
SEM images of (**a**) SL-CNTs used for length measurements, (**b**) magnified image of mapped out section in (**a**), (**c**) SL-CNTs on different particles, (**d**) magnified image of mapped out section in (**c**).

The morphology of the SL-CNTs after microwave treatment is presented in Figure 2, showing a dramatic change in the morphology of the CNTs after treatment at 1200 °C, 1300 °C, and 1400 °C. Figure 2a shows that significant changes in the length and morphology of the CNTs took place after treatment at 1200 °C. However, at this temperature, the CNTs were still twisted and randomly entangled. With further increase in the treatment temperature to 1300 °C and 1400 °C, CNTs became straight and longer (Figure 2b,c). An average diameter of approximately 300 nm and lengths ranging from 0.7–2 mm were measured. To our knowledge, this is the longest CNT synthesized directly from biomass without the addition of an external catalyst. The CNT’s contour length and endpoints were traced using the freehand tool in ImageJ to measure long twisted CNTs. The SEM images used for measurement and the obtained length measurements are shown in Figure 3 and Table 2, respectively.

The EDS results of the SL-CNTs at 1400 °C (Figure 2d) revealed Si, Ca, Mg, Fe, Na, and K [39]. These results suggested that the presence of inherent metallic species in biomass may have catalyzed the growth of the CNTs under high-temperature microwave treatment conditions [24].

**Figure 4:**
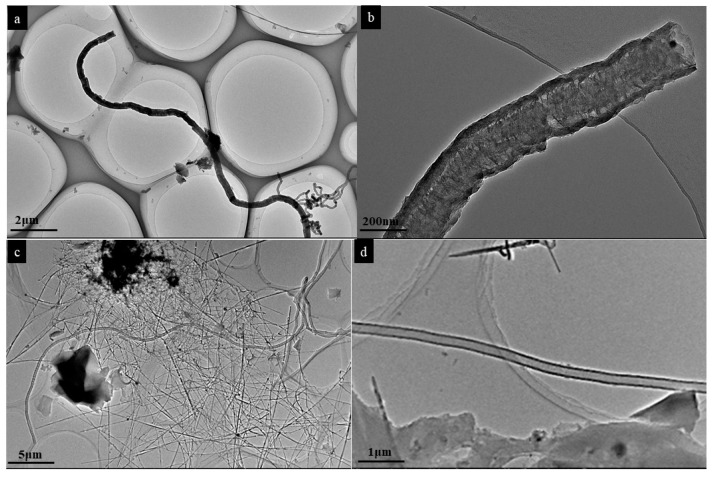
HRTEM images of CNTs after microwave treatment: (**a**) 1200 °C, (**b**) magnified image of (**a**), (**c**) 1300 °C, (**d**) magnified image of (**c**).

The HRTEM analysis of the SL-CNTs after microwave treatment at 1200 °C and 1300 °C is shown in Figure 4. Some CNTs exhibited curved morphology (Figure 4a), while others had a vertical structure (Figure 4d). This may have resulted from the interaction between microwave irradiation and CNTs at high temperatures. The thermal effect of microwave treatment at elevated temperatures induces changes in the curved structure of CNTs in the presence of inherent inorganic matter in CNTs, thereby changing the morphology.

**Figure 5:**
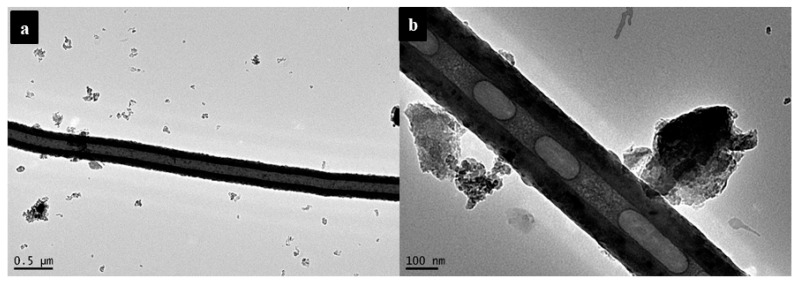
HR-TEM images of SL-CNTs synthesized at 1400 °C: (**a**) and (**b**) show different magnifications.

Figure 5 shows the hollow structure of CNTs. The *d*-spacing value of 0.32 nm was calculated for CNTs, slightly lower than that of natural graphite (0.335 nm). Figure 5b shows that thick graphene layers formed bridges between the walls of CNTs. The changes in the structure of CNTs were attributed to the high mobility of carbon atoms at elevated temperatures [40,41]. This implies that structural change in SL-CNTs can be obtained under microwave treatment at high temperatures even though the CNTs initially had a different structure at lower temperatures.

### 3.2. Crystalline Structure of CNTs

**Figure 6:**
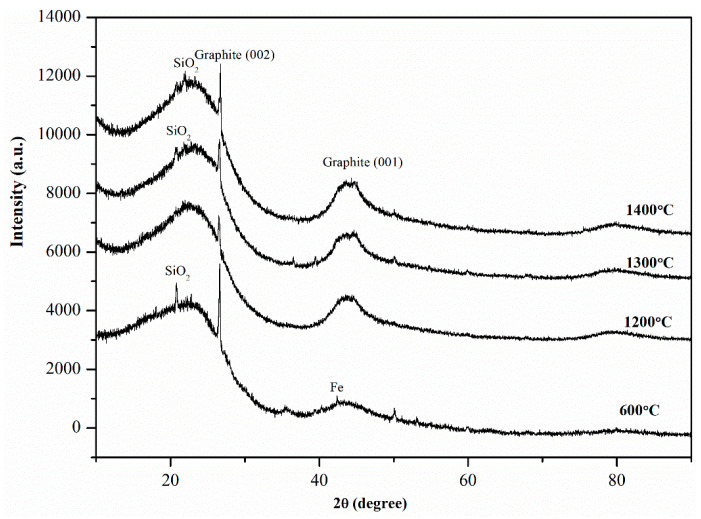
X-ray diffraction spectra of CNTs after microwave treatment at different temperatures.

The typical peaks for CNTs in XRD spectra are generally detected at 2θ = 26° and 2θ = 43°, which illustrate the graphite (002) and (100) reflections, respectively [24]. Figure 6 shows two prominent peaks at 2θ = 26.3° and 2θ = 43.2° in the XRD spectra of CNTs after microwave treatment, suggesting that the CNTs possessed crystalline structure. The peak at 2θ = 43.2° was not detected at 600 °C. However, after high-temperature microwave treatment, the peak became prominent. This is attributed to the increased carbon order of CNTs and a graphitic structure at higher temperatures. Si and Fe were observed in the CNT sample as their corresponding peaks were detected in the XRD spectra.

To quantify the yield of SL-CNTs, the crystallinity of biochar samples was analyzed using Rietveld refinement quantitative analysis. The approach reported in [35] was used in this study. The results showed that the yield of CNTs increased from 9.81 wt.% at 600 °C to 17.32 wt.%, 23.54 wt.%, and 24.89 wt.% at 1200 °C, 1300 °C, and 1400 °C, respectively. These results imply that the high-temperature microwave treatment substantially enhanced the yield of CNTs without the need for additional catalysts or carbon sources to be used.

### 3.3. Changes in the Carbon Order of CNTs

**Figure 7:**
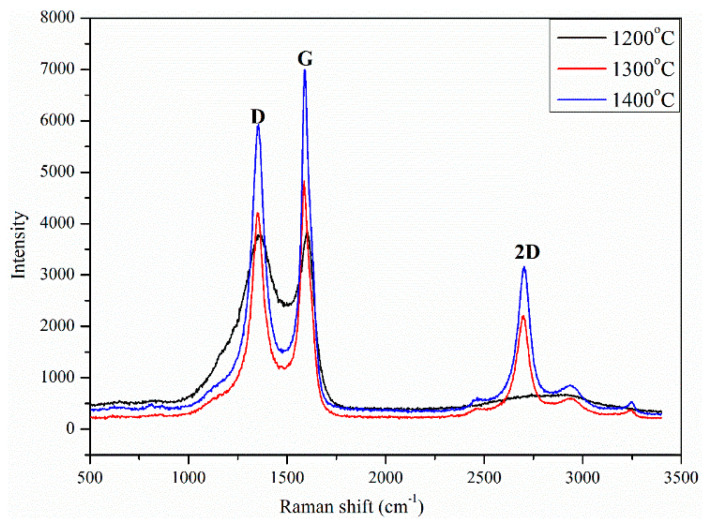
Raman spectra of SL-CNTs as a function of synthesis temperature.

Raman spectra of the CNTs before and after high-temperature treatment is shown in Figure 7. Raman spectroscopy technique was used for investigating the degree of carbon order, the presence of defects, and the degree of crystallization [42,43]. The first-order Raman spectra of all samples displayed the typical bands that correlate with carbonaceous structures. The G band at 1590 cm^−1^ corresponds to the stretching mode in graphite planes associated with the sp^2^ bond. The D band at about 1360 cm^−1^ correlates with disorder and defects in highly ordered carbonaceous materials or graphite crystals [44,45]. As seen in Figure 6, the G band of the SL-CNTs increased with temperature, while the D band showed an opposite trend, implying that the degree of carbon order was increased at higher temperatures [46]. The D band intensity ratio to G band (*I_D_/I_G_*) is a significant parameter for studying graphite-like or crystalline carbon structures. It indicates the degree of carbon order; a high ratio indicates a high defect in the carbon structure, while a lower ratio signifies a high carbon order and lower defect in the carbon structure [47]. The *I_D_/I_G_* ratio is commonly used to evaluate the degree of graphitization in carbon materials like CNTs. The *I_D_/I_G_* ratio of CNTs decreased from 0.97 at 1200 °C to 0.87 at 1300 °C, finally to 0.84 at 1400 °C. These results show that the carbon order and graphitization degree of SL-CNTs increased at elevated temperatures.

To better understand the alteration in the microstructure of CNTs after microwave treatment, further analysis was carried out by deconvoluting the Raman spectra of the SL-CNTs into five bands by adopting the Gaussian function method. The additional bands at 1196 cm^−1^ (R1), 1635 (R2) cm^−1^, and 1515 cm^−1^ (R3) were considered. Aromatic carbon, aromatic ethers, and alkyl carbon bonds are linked to the R1 band [48]. The R2 band corresponds to the disordered structures of CNTs. The R3 band represents the functional groups in carbonaceous materials [49]. The area proportions of the deconvoluted Raman spectra of the SL-CNTs after microwave treatment are shown in Table 3. Noticeable changes were observed in the area of different bands with changes in temperature. An increase in the G band was evident at higher temperatures, indicating the transformation of amorphous carbon to graphitic carbon. The decrease in the R1 and R3 bands indicated the removal of organic structures and functional groups from the structure of CNTs at elevated temperatures under microwave treatment conditions.

**Figure 8:**
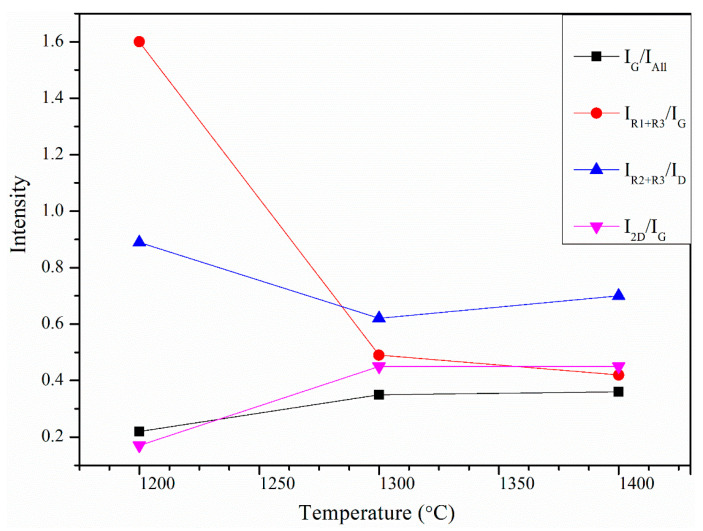
Band area ratio variations from the curve-fitting analysis of SL-CNTs Raman spectra as a function of temperature.

The integrated band area ratios were defined and used to better understand the changes in the carbon structure of SL-CNTs, and the results are shown in Figure 8. The *I_R2_+I_R3_*/*I_D_* ratio studied the formation of aromatic clusters during the growth of SL-CNTs. The amorphous carbon clusters containing 3–5 aromatic rings are represented by *I_R2_+I_R3_*, while *I_D_* reflects aromatic cluster development with more than six rings [50]. Figure 8 shows that the *I_R2_+I_R3_/I_D_* ratio of SL-CNTs decreased from 0.89 at 1200 °C to 0.70 at 1400 °C, indicating the transformation of small aromatic systems to larger aromatic clusters, which in turn increased the carbon order of SL-CNTs. The monotonous increase in the *I_G_/I_All_* ratio with temperature suggested that the graphitic structure of SL-CNTs was enhanced at higher temperatures. The *I_R1_+I_R3_/I_G_* ratio of SL-CNTs decreased from 1.60 at 1200 °C to 0.42 at 1400 °C. These results imply the removal of functional groups and the development of more ordered structures in SL-CNTs.

Figure 8 also shows that the intensity of the 2D band at 2678 cm^−1^ increased with temperature. This band is known as the overtone of the D band in the second-order Raman spectra. The shape, intensity, and frequency of the 2D band are affected by the chemical structure of CNTs and the number of carbon layers in the CNTs. The *I_2D_/I_G_* values of less than one in the SL-CNTs (Figure 8) indicated the development of multi-layered graphitic structures.

### 3.4. Mechanism of Formation of SL-CNTs

Based on the above results, the mechanism of the formation of SL-CNTs hinges on three factors, namely the catalytic role of the inherent minerals in the biomass, the unique effect of microwave heating, and the microwave treatment temperature. Inorganic matter in biomass can influence its decomposition behavior and the yield and composition of products [39,51,52]. We infer that the growth of SL-CNTs is a two-stage mechanism: (1) the formation of CNTs at low temperatures and (2) modification of structure and further growth under high-temperature microwave treatment. Based on the microwave heating mechanism, microwave energy is converted into heat when it penetrates the particle. The heat continues to accumulate in the particle core, leading to the formation of a temperature gradient. Due to internal pressure buildup, the generated volatile matter moves from the hot center to the particle’s lower-temperature surface.

The first stage of this mechanism has been reported in our previous study [35]. Briefly, volatiles generated during the thermal decomposition of biomass form droplets on the particle’s surface. The continuous release of volatiles, via self-extrusion from the nanopores, led to the growth of the CNTs. The EDS analysis showed minerals on the body and tip of the CNTs (Figure 1). Since no external catalyst was used, these elements are confirmed to originate from cellulose.

In the second stage, when the biochar containing CNTs was subjected to secondary heating under high microwave treatment at elevated temperature, pyrolysates from the biochar were generated. It served as a carbon source. These pyrolysates generated from the low-temperature biochar were the volatiles remaining in the char after pyrolysis (approximately 17 wt.%). The volatiles left in the char were high molecular weight compounds that failed to generate enough vapor pressure to be released from the biomass [53]. These volatile materials were mainly composed of polyaromatic hydrocarbons (PAH), which acted as carbon sources for the growth SL-CNTs at elevated temperatures. The growth of SL-CNTs can occur through more than one route. We propose two possible routes of formation:

I. The CNTs formed at low temperature act as templates for the growth of the SL-CNTs. The tip of the CNT is known to be very reactive [54]. The volatiles released from the biochar at high-temperature form PAH, which, in turn, adds graphene layers to the CNT tips, leading to the growth of the SL-CNTs. This agrees with the TEM results, which revealed an increase in the diameter after high-temperature treatment.
II. Through active sites in the char, which were activated by minerals or metals during high-temperature treatment. Char can be catalyzed by a trace amount of minerals that originate from the biomass. The minerals present in the char, notably Fe, produced a catalytic effect in the CNTs’ growth. From previous investigations carried out by other researchers, CNTs can form, or even grow, in the presence of a meagre percentage of Fe catalyst [55]. The XRF analysis revealed at least 11.62 wt.% of Fe was present in the ash, as shown in Table 1. Due to the microwave’s unique heating, at high temperature, volatiles released encapsulate the metal particle (Fe) and elements present in the biomass char via surface or sub-surface diffusion. This process initiates the growth process by assembling carbon radicals into graphene around the element clusters, leading to the increased growth of SL-CNTs. This corresponds to the EDS analysis, where Fe and Si were observed on the SL-CNTs. The Fe was not visible in the XRD at a higher temperature because it was in low quantity and had high dispersion. Hence XRD could not identify some of its phases.

The change in the morphology of CNTs from curvy to straight may be attributed to the structural modification at high temperatures. Mobility of carbon atoms leads to re-orientating the carbon structure, and changes in its physical properties can occur at elevated temperatures [56,57]. In addition, the presence of the Si may affect the morphology changes, as Si is known to contribute to the formation of the straight-like morphology of nanotubes at elevated temperatures [39].

Table 4 shows the comparison between the SL-CNTs generated from this study in relation to those of SL-CNTs from other methods in the literature discussed in the Introduction. The main difference is the carbon source and the methods of synthesis. Biomass was used in this study, while other methods used gases from fossil fuels. In the synthesis method, microwave pyrolysis without a catalyst was used in this study, while other methods include CVD, laser ablation, arc discharge method, etc., with the aid of metallic catalysts. Structural defects were observed in the SL-CNTs synthesized in this study, as well as those reported in literature.

### 3.5. Implications of the Novel SL-CNTs Production Method

Although carbon nanotubes are being used widely in various technological fields, they still have some significant drawbacks that hinder their full potential due to their small dimensions and the lifespan of added catalysts. Previously, SL-CNT formation was dependent on the catalyst’s lifetime. The electrical and mechanical properties of CNTs can only be harnessed if the CNTs are long and continuous for some applications. This novel method of producing SL-CNTs is simple, safe, and environmentally friendly and an ethical approach towards synthesizing cleaner, high purity, and low-cost SL-CNTs from biomass without the need for an additional catalyst or carbon source under microwave treatment. Therefore, the method can provide a convenient means of producing selective CNT morphology, leading to the mass production of SL-CNTs on an economic basis. SL-CNTs pave the way for novel opportunities for capitalizing on CNT properties over large length scales. The SL-CNTs formed are more durable and possess strong electrical and mechanical properties, making them valuable for an increasingly large number of applications in the field of nanotechnology, material science, and even modern-day living. The advantages of SL-CNTs over their shorter counterparts include enhanced suitability for electrical conduction, multifunctional composites, sensors, and nanoelectronics. SL-CNTs offer a combination of flexibility, electrochemical reactivity, strength, electrical conductivity, and porosity, which will pave the way for a new era of technological advancement.

## 4. Conclusions

Super-long carbon nanotubes (SL-CNTs) with lengths of up to 2 mm were successfully synthesized from cellulose biomass via microwave treatment without any catalyst or external carbon source. A low Raman I_D_/I_G_ ratio of 0.84 after microwave treatment at 1400 °C was obtained, indicating an increase in the carbon order in the SL-CNTs. The yield of SL-CNTs reached 26.89 wt.% at 1400 °C. The inherent inorganic matter in biomass was postulated to have functioned as a catalyst to facilitate the growth of the SL-CNTs. The carbon supply source for the SL-CNTs under microwave treatment was the aromatic hydrocarbons generated from the biochar. The change in the morphology and structure of CNTs after microwave treatment can be used to control the quality and structure of CNTs from cellulosic biomass. The method of SL-CNT synthesis presented here is a simple but sustainable method for the synthesis of nanotubes from renewable biomass without the use of any catalyst or external carbon source. Hence, this is a promising approach for large scale production of superlong carbon nanotubes that should find applications in various industries.

## Figures and Tables

**Table 1:** Chemical composition of PKS cellulose ash obtained by XRF analysis.

Biomass Element	Weight (%)
Mg	1.80
Al	3.95
Si	72.10
P	0.50
K	2.10
Ca	3.60
Fe	11.62

**Table 2:** Length measurements of SL-CNTs from ImageJ analysis.

Mean	StdDev	Min	Max	Length (mm)
87.82	36.55	40.08	194.35	1.06
93.29	30.42	46.14	183.45	0.77
79.10	23.81	41.00	180.00	1.27
89.94	37.62	33.26	226.56	0.91
79.65	42.55	30.49	255.00	1.80
70.34	39.25	29.13	252.49	2.23

**Table 3:** Deconvoluted peaks area fraction as a function of pyrolysis temperature.

Temperature (°C)	Area Proportion				
	R1	D	R3	R2	G
1200	0.23	0.35	0.23	0.06	0.11
1300	0.17	0.32	0.20	0.08	0.18
1400	0.18	0.30	0.22	0.08	0.20

**Table 4:** Comparison between the SL-CNTs synthesized in this study and literature results.

Synthesis Process/Parameters	Synthesized SL-CNTs in This Study	SL-CNTs Synthesized from Other Methods
Carbon source	Volatiles in biomass	Gases derived from fossil fuels (methane, ethane, acetylene, etc.)
Catalyst	No catalyst	metallic catalysts (Ni, Mo, Fe, Co, etc.)
Process	Microwave synthesis	Laser ablation, Arc discharge, CVD, CCVD, PECVD
Temperature	600–1400 °C	700–2500 °C
Time	30 min	Ranges from 1–10 h
Length (mm)	0.7–2	1–5
Morphology	Ordered carbon structure with some defect	Ordered carbon structure with some defect

## Data Availability

Data presented in this study are available in this article.
